# Undifferentiated Rhabdoid Carcinoma of the Gastrointestinal Tract: A Rare and Aggressive Malignancy

**DOI:** 10.7759/cureus.79237

**Published:** 2025-02-18

**Authors:** Justin M Hsieh, Zara Summers, Shinn Yeung

**Affiliations:** 1 Department of Surgery, Greenslopes Private Hospital, Brisbane, AUS

**Keywords:** gastrointestinal mass, malignant rhabdoid tumors, rare malignancy, rhabdoid features, undifferentiated carcinoma

## Abstract

Undifferentiated carcinoma with rhabdoid features is an exceptionally rare malignancy within the spectrum of malignant rhabdoid tumors. We report a case of a healthy 51-year-old male presenting with an abdominal mass, ultimately diagnosed as an undifferentiated carcinoma with rhabdoid features. Histopathologic examination and immunohistochemistry revealed the distinct morphological and molecular characteristics of this aggressive tumor. This case adds to the limited published literature on this rare presentation and highlights the need for further investigation into its pathogenesis and optimal management.

## Introduction

Undifferentiated carcinoma with rhabdoid features in the gastrointestinal (GI) tract is a rarely reported entity within the malignant rhabdoid tumor (MRT) family. These tumors are associated with highly aggressive behavior and poor prognosis, with reported involvement in various organs such as the heart, nervous system, breast, and GI tract [1,2]. The exact incidence of MRTs in the GI tract currently remains poorly defined due to their rarity. There were no incidences of MRTs found in the small intestine, and very few incidences have been reported in the colon [2]. Histologically, these tumors are characterized by large, eccentrically placed nuclei, prominent nucleoli, and cytoplasmic aggregates of intermediate filaments [3,4]. Immunohistochemically, they typically co‐express both epithelial and mesenchymal markers [4]. Despite their unique phenotypic characteristics, data on optimal management and outcomes remain limited due to limited reports on the tumor. Here, we report the clinical course of a 51-year-old patient diagnosed with undifferentiated carcinoma with rhabdoid features of the jejunum.

## Case presentation

**Figure 1:**
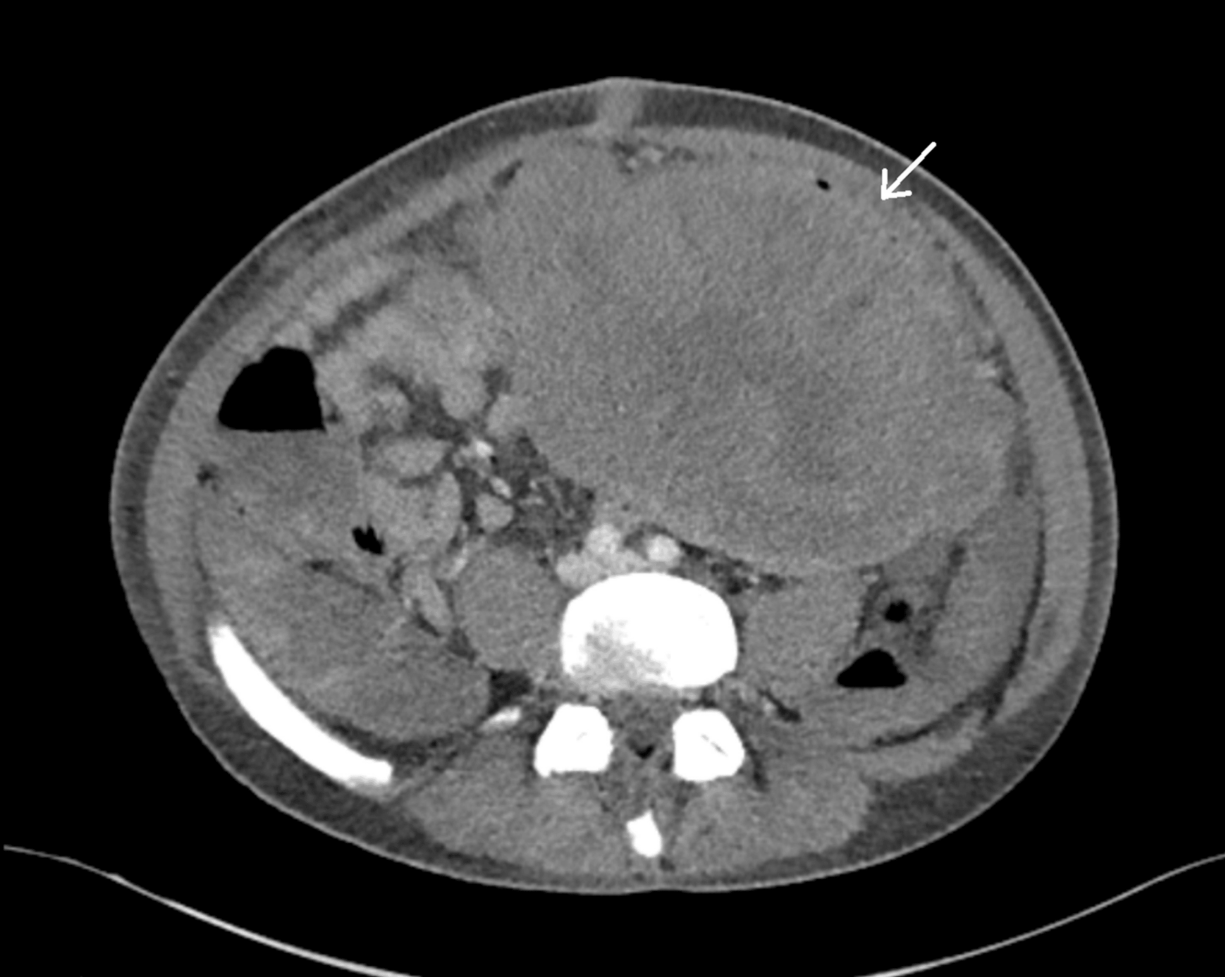
Axial CT scan of the MRT White arrow indicating the intra-abdominal tumor CT, computed tomography; MRT, malignant rhabdoid tumor

**Figure 2:**
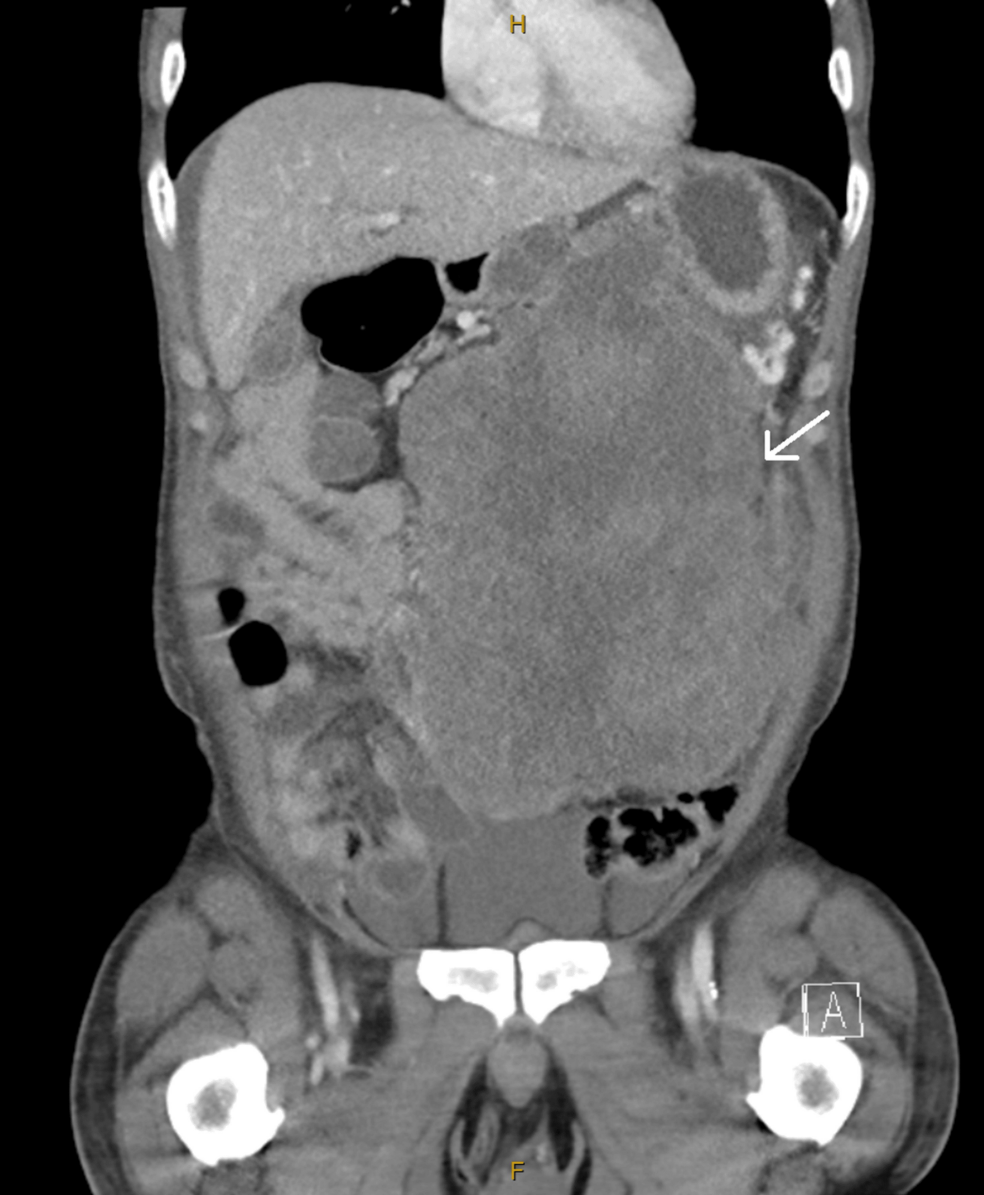
Coronal CT scan of the MRT White arrow indicating the large intra-abdominal tumor CT, computed tomography; MRT, malignant rhabdoid tumor

**Figure 3:**
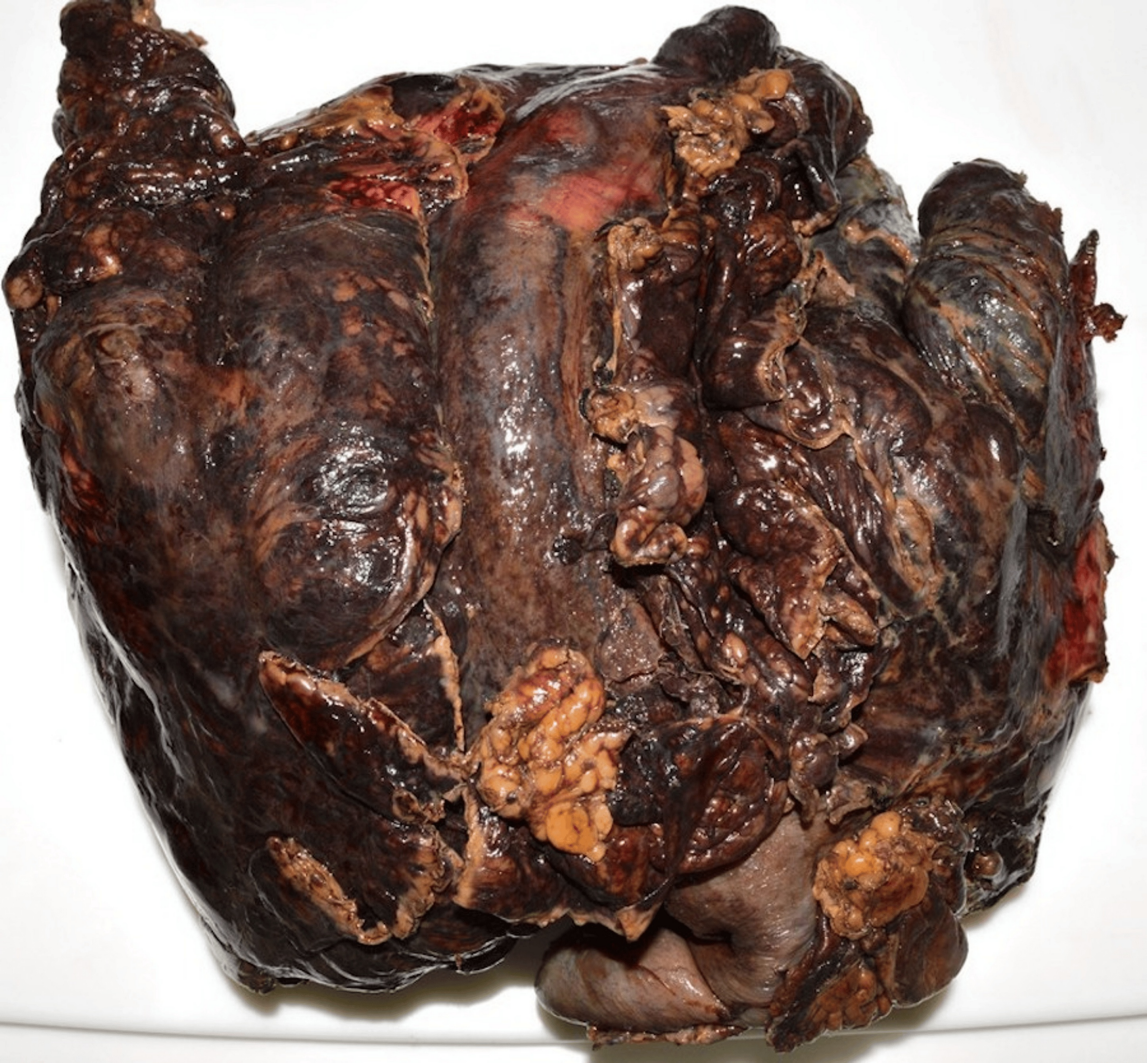
Specimen of the MRT after en bloc resection MRT, malignant rhabdoid tumor

A 51-year-old male with no past medical history presented to his primary care doctor with several months of intermittent generalized abdominal pain. The patient was otherwise well, with no other symptoms apart from his pain. A computed tomography (CT) was ordered, revealing a large abdominal mass presumed to be a jejunal gastrointestinal stromal tumor (GIST) (Figures 1, 2). Pre-operatively, there were no CT findings suggesting of active bleeding or bowel perforation, and the patient elected for a diagnostic laparoscopic surgery. However, intraoperatively, the diagnostic laparoscopy revealed a large, perforated tumor in the abdomen with active bleeding, prompting an emergent open en bloc resection and small bowel anastomosis after discussion with the patient’s next of kin (Figure 3). At the time of the operation, the tumor was completely removed macroscopically, with no visible signs of peritoneal disease. The patient recovered well in the initial postoperative phase and was due to commence on adjuvant chemotherapy. However, before commencing chemotherapy, he subsequently experienced a large bowel obstruction from multiple peritoneal metastases, necessitating an emergency stoma placement and palliative care input. Despite recovering well from his stoma placement, the patient passed away within three months of initial CT diagnosis from rapidly worsening tumor burden.

**Figure 4:**
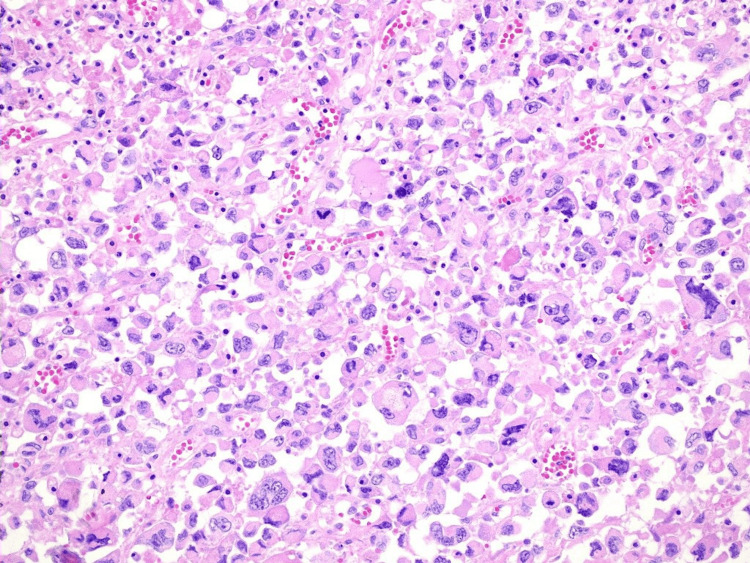
20x hematoxylin and eosin (H&E) stain showing epithelioid cells with eosinophilic cytoplasm, pleomorphic nuclei, and prominent nucleoli

**Figure 5:**
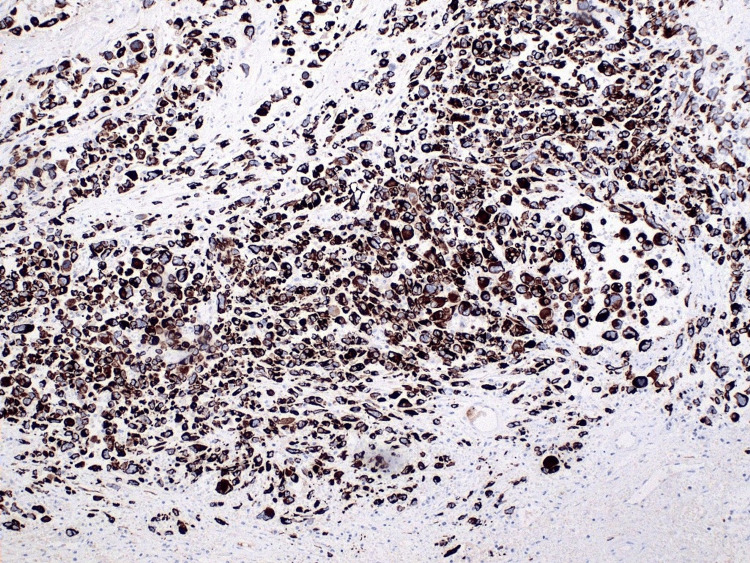
Immunohistochemistry showing strongly positive cytokeratin AE1/AE3 stains

Histological examination revealed an undifferentiated carcinoma with rhabdoid features, characterized by epithelioid cells with eosinophilic cytoplasm, pleomorphic nuclei, and prominent nucleoli, situated within the subserosa and mesentery of both small and large bowels (Figure 4). The cells also displayed significant cytological atypia and a high mitotic rate within the hemorrhagic and necrotic stroma. Immunohistochemistry showed positive expression for cytokeratin AE1/AE3 and CAM5.2 and lack expression of a broad spectrum of other epithelial and melanocytic markers (Figure 5). The Ki67 proliferation index was 60%, with positive vimentin and loss of INI1 staining. The negative results for Lynch syndrome markers further confirm the sporadic nature of this malignancy. This pattern, coupled with the presence of the BRAF V600E mutation and normal expression of mismatch repair (MMR) proteins indicating MMR proficiency, supports the diagnosis of an undifferentiated carcinoma with rhabdoid features. This is an exceptionally rare malignancy characterized by the unique morphology and immunohistochemistry.

## Discussion

MRTs are exceedingly rare, especially within the GI tract, and have unique clinical and pathological characteristics. A 2019 literature review by Kojima et al. reported only 28 cases of MRT in the colon from 1993 to 2019, highlighting both the rarity of this tumor type and its aggressive course [1]. The vast majority of these cases (24 out of 28) presented with metastatic disease, reflecting a highly malignant phenotype that correlates with a median survival of only three months, findings that closely mirror our patient’s short survival interval [1,5]. Moussaly and Atallah, in a 2015 literature review, also described a mean onset age of 70 years for this entity, with an equal sex distribution [6]. Common presenting features include nonspecific abdominal pain, a palpable abdominal mass, and GI bleeding [6]. Although our patient did not have any GI bleeding or macroscopic metastatic disease, other clinical features mirror previous case reports.

Since insights into rhabdoid carcinomas come from only a small number of cases, it is commonly misdiagnosed with several other malignancies in the GI tract due to their histological similarities. One key differential diagnosis is sarcomatoid carcinoma, which often shares the characteristic pleomorphic morphology with rhabdoid tumors. However, sarcomatoid carcinoma typically lacks the prominent nucleoli and rhabdoid inclusions seen in MRTs [5,7]. Additionally, sarcomatoid carcinoma often lacks the immunohistochemical co‐expression of epithelial markers, cytokeratin AE1/AE3, and mesenchymal markers, which are typical in MRTs [7]. Another potential differential is diffuse large B-cell lymphoma, which can present with large cell morphology and lymphocytic infiltration similar to that of MRTs. However, lymphoma cells are usually positive for CD20 and negative for cytokeratin, while MRTs exhibit cytokeratin positivity along with mesenchymal markers such as vimentin [7]. Melanoma, another possible differential, may also be considered due to its pleomorphic appearance, but it typically shows positive staining for melanocytic markers, which are absent in MRTs [7]. Distinguishing these entities is crucial, as treatment strategies and prognoses vary widely between different diagnoses.

Key characteristics include that MRTs are broadly classified into two categories based on histological composition. The “pure” form consists exclusively of undifferentiated carcinoma cells possessing rhabdoid morphology, whereas the “composite” form includes areas of both undifferentiated rhabdoid cells and more differentiated malignant components [3,7]. Although the presence of more differentiated elements in the composite form might suggest a less aggressive biological behavior, current evidence does not clearly establish distinct differences in clinical presentation, overall outcomes, or recommended treatment approaches between these two subtypes. Both remain highly aggressive with poor prognoses, and no standardized management guidelines stratify therapy for either subtype. In our patient, the histologic findings of mixed differentiation suggest a “composite” lesion, although it is unclear whether this distinction would have significantly altered therapeutic decisions or prognosis in the context of such an aggressive malignancy.

Genetic and immunohistochemical findings in MRTs frequently include alterations in tumor suppressor genes, such as SMARCB1 (which encodes INI1), and mutations in oncogenes, such as BRAF [1,3,4]. Loss or reduction of INI1 expression is a common feature in MRTs of various origins [1]. Additional mutations, such as BRAF V600E, have been documented in rhabdoid carcinomas of the colon [3,5,8]. In our patient, MMR protein expression (MSH2, MSH6, MLH1, PMS2) remained intact, aligning with the sporadic nature of the tumor and excluding Lynch syndrome-associated lesions. Although KRAS phenotypes were not systematically evaluated, prior reports have highlighted a wild‐type KRAS status in many tumors harboring BRAF mutations [3,5,8].

From a histopathological standpoint, MRT cells display abundant eosinophilic cytoplasm, large vesicular or round nuclei, prominent nucleoli, and hyaline, “rhabdoid” cytoplasmic inclusions [1,3,9]. Despite their sarcoma‐like morphology, these cells often co‐express epithelial (cytokeratin AE1/AE3) and mesenchymal (vimentin) markers and are typically negative for muscular lineage proteins [10]. Clinically, MRTs of the colon have a particularly poor prognosis, with some reports citing a mortality rate of up to 75% within six months following diagnosis [8]. In one meta‐analysis, Horazdovsky et al. observed a potential survival benefit with combined surgery and actinomycin for patients with MRT, although this finding remains far from definitive [11]. Due to its rarity and high mortality, a standardized treatment algorithm is currently lacking.

In practice, the exceedingly high mortality and frequent presentation with metastatic or rapidly progressive disease cast doubt on the benefit of aggressive surgical intervention, and a palliative approach. Nonetheless, in the present case, surgical resection was pursued because the tumor was initially presumed to be a GIST and was found to be perforated upon diagnostic laparoscopy, an acute abdomen necessitating urgent operative management. Had the rhabdoid phenotype been recognized preoperatively, the decision to operate might have been more complex, especially given the typically poor prognosis associated with these tumors.

Ultimately, undifferentiated carcinoma with rhabdoid features in the GI tract demands heightened clinical suspicion and thorough pathologic evaluation. The aggressive course of our patient’s disease, manifesting rapid progression, early metastasis, and poor outcomes, illustrates the characteristic lethality of this entity, consistent with the limited data available [12]. In light of the scattered case reports and small case series thus far, future investigations will be important to clarify the underlying molecular pathogenesis, identify more effective treatments, and ultimately improve survival for patients affected by this rare and aggressive malignancy.

## Conclusions

Undifferentiated carcinoma with rhabdoid features in the colon is a rare and aggressive malignancy with limited understanding and treatment options. So far, our understanding of this condition has come from a handful of isolated case reports and studies that lack data. This case contributes valuable information to the scarce literature on this topic, emphasizing the need for in-depth studies to enhance our knowledge and improve outcomes for patients with this rare malignancy.
